# COLOMBOS v2.0: an ever expanding collection of bacterial expression compendia

**DOI:** 10.1093/nar/gkt1086

**Published:** 2013-11-08

**Authors:** Pieter Meysman, Paolo Sonego, Luca Bianco, Qiang Fu, Daniela Ledezma-Tejeida, Socorro Gama-Castro, Veerle Liebens, Jan Michiels, Kris Laukens, Kathleen Marchal, Julio Collado-Vides, Kristof Engelen

**Affiliations:** ^1^Department of Mathematics and Computer Science, University of Antwerp, B-2020 Antwerp, Belgium, ^2^Biomedical Informatics Research Center Antwerp (biomina), University of Antwerp/Antwerp University Hospital, B-2650 Edegem, Belgium, ^3^Department of Computational Biology, Research and Innovation Center, Fondazione Edmund Mach, San Michele all'Adige, Trento (TN) 38010, Italy, ^4^Department of Microbial and Molecular Sciences, KU Leuven, Leuven B-3001, Belgium, ^5^Centro de Ciencias Genómicas, Universidad Nacional Autónoma de México, Cuernavaca, Morelos 62210, Mexico, ^6^Department of Plant Biotechnology and Bioinformatics, Ghent University, Gent 9052, Belgium and ^7^Department of Information Technology, IMinds, Ghent University, Gent 9052, Belgium

## Abstract

The COLOMBOS database (http://www.colombos.net) features comprehensive organism-specific cross-platform gene expression compendia of several bacterial model organisms and is supported by a fully interactive web portal and an extensive web API. COLOMBOS was originally published in PLoS One, and COLOMBOS v2.0 includes both an update of the expression data, by expanding the previously available compendia and by adding compendia for several new species, and an update of the surrounding functionality, with improved search and visualization options and novel tools for programmatic access to the database. The scope of the database has also been extended to incorporate RNA-seq data in our compendia by a dedicated analysis pipeline. We demonstrate the validity and robustness of this approach by comparing the same RNA samples measured in parallel using both microarrays and RNA-seq. As far as we know, COLOMBOS currently hosts the largest homogenized gene expression compendia available for seven bacterial model organisms.

## INTRODUCTION

COLOMBOS, originally the acronym for ‘COLections Of Microarrays for Bacterial OrganismS’, hosts several large expression compendia derived from high-throughput expression experiments with an explicit focus on bacterial organisms (1). The expression experiments available in COLOMBOS are derived from public resources, such as the Gene Expression Omnibus (2) or ArrayExpress (3) repositories, but the actual data originates from a reanalysis starting from the raw hybridization intensities for microarrays, or short read sequences for RNA-seq, using a consistent and robust normalization pipeline with stringent quality controls at each step. This procedure yields high quality expression compendia that can directly integrate high-throughput expression data from different technological platforms. It is unique in this respect, as gene expression compendia in general either only rely on experiments from a single technological platform to directly integrate data, or indirectly integrate data from cross-platform experiments (so that only the results of separate analyses on the individual experiments are integrated, not the actual measurements). The expression data contained within the database have been linked to a manually curated, standardized condition annotation and ontology created specifically for the COLOMBOS compendia, as well as heterogeneous gene annotation information, such as metabolic pathways or transcriptional regulation, from other public databases. Both the condition and gene annotation provide a lot of flexibility when querying the database and analysing the returned results through a suite of expression exploration, analysis and visualization tools. Programmatic access to the database has now also been made available through a REST web service and as an R package.

The usage of the COLOMBOS database for scientific research has been very diverse. Common operations include starting from a set of known genes to find the conditions where they are (co)-expressed (4) or to identify additional co-expressed genes (5–7). These types of analyses can be readily accomplished using the tools available within the COLOMBOS web interface (4,6). The functionalities of the interface are designed for users to ‘play around’ with the compendia to make the most out of the data given the biological question they are interested in. They are encouraged to try different types of search queries based on genes or conditions, find additional (anti-)co-expressed genes, generate clusters to separate disjoint expression profiles, explore the overlap between multiple queries and potentially combine them, etc. There are several detailed tutorials on the website illustrating how concrete examples of conceptually different biological questions could be handled through the COLOMBOS interface. The compendia are also available for download in their entirety for application of stand-alone tools, allowing usage of COLOMBOS data within the greater scope of systems biology (8–11) by, e.g. creating co-expression networks directly from the expression data (12,13) or by using entire expression compendia for transcriptional regulatory network inference (14). The formalized condition contrast annotation found in COLOMBOS has made it ideal for linking gene expression changes to the underlying causal factors, such as activation of transcription regulators by effectors (15) or genomic mutations (16).

## DATA CONTENT UPDATE

### New and updated compendia

An overview of the data content of the seven species’ gene expression compendia can be found in Table 1. The most defining characteristics are the number of genes and number of contrasts as these give an indication of the size of the compendium expression data matrix. The rows of a compendium matrix correspond to the known genes of the organism in question. We refer to the columns as ‘condition contrasts’ because they do not represent single experimental conditions or samples, but in fact always represent the difference between a test and reference condition (the expression values themselves are calculated as expression logratios). In brief, the three compendia that were made available with the original publication (for *Escherichia coli*, *Bacillus subtilis* and *Salmonella enterica* serovar Typhimurium) have been greatly expanded with new experiments that have been published in the meantime. For example, the *E. coli* compendium now includes data for over 2400 measured conditions, for over 1000 contrasts more that was available in the previous version. The gene annotation from external databases incorporated for these species [e.g. RegulonDB (17), BioCyc (18) and EcoCyc (19)] has been updated to the latest version. We have also built compendia for four new species, all with strong biomedical relevance: *Streptomyces coelicolor*, *Pseudomonas aeruginosa*, *Mycobacterium tuberculosis* and *Helicobacter pylori*. Each of these four new compendia features its own unique standardized condition contrast annotations, as a single condition may have widely different effects in different species, and these annotation terms have been manually assigned to each condition contrast within these compendia. Gene annotation data from public resources, such as BioCyc (18) and UniProt-GOA (20), have been integrated to allow flexible data querying in the same manner as for the three original species. In addition, some species-specific annotation information was also included, such as the recently published transcriptional regulatory network of *M. tuberculosis* (21). For each of the seven organisms, recent RefSeq genome files [from NCBI (22), see Table 1] were used to construct unique lists of genes, which correspond to the rows of the final compendia expression matrices. Microarray probes were mapped to these lists of genes in a platform-specific manner, and then data derived for the corresponding experiments were processed using the homogenization and normalization pipelines as described in the original COLOMBOS publication (1), where various quality metrics for each array (intensity distributions, MA plots, robust estimates of error noise, etc.) were evaluated prior to the inclusion of an experiment in the compendia. This ensures that the final compendia only include high quality homogenized expression data that result from a consistent processing pipeline.

**Table 1.** Overview of the data available in COLOMBOS for the seven expression compendia

	*E. coli*	*B. subtilis*	*S. enterica* srv. Typhimurium	*S. coelicolor*	*P. aeruginosa*	*M. tuberculosis*	*H. pylori*
Genome refseq id	NC_000913.2 (4 March 2013)	NC_000964.3 (20 August 2012)	NC_003277.1, NC_003197.1 (3 April 2013)	NC_003888.3, NC_003903.1, NC_003904.1 (23 May 2013)	NC_002516.2 (23 May 2013)	NC_000962.3 (15 February 2013)	NC_000915.1 (21 December 2012)
Number of genes	4321	4167	4560	8239	5647	4068	1617
Number of contrasts	2470	723	914	371	482	549	122
Missing values	3.11%	4.54%	6.42%	7.35%	1.53%	2.81%	3.09%
Source DB	GEO, AE	GEO	GEO, AE	GEO, AE	GEO	GEO	GEO
Samples	3435	964	1594	546	511	1095	244
Experiments	131	21	37	9	29	11	7
Platforms	39	21	22	7	2	11	4
External DBs							
Pathway	EcoCyc	BioCyc	BioCyc	BioCyc	BioCyc	BioCyc	BioCyc
Regulon	RegulonDB	BsubCyc,DBTBS				Galagan *et al.* (21)	
Operon	RegulonDB	BioCyc	BioCyc	BioCyc	BioCyc	BioCyc	BioCyc
GO	UniProt GOA	UniProt GOA	UniProt GOA	UniProt GOA	UniProt GOA	UniProt GOA	UniProt GOA

### Incorporation of RNA-seq data

The expression compendia were originally built solely from microarray data, but the backend compendia tools were designed from the ground up to be future proof. In the meantime, we have implemented pipelines that allow us to incorporate RNA-seq data. As RNA-seq data for bacterial species are still relatively scarce, only three of COLOMBOS’ compendia currently include it (*E. coli*, *S. enterica* serovar Typhimurium and *M. tuberculosis*), but this will for sure change in the near future as more RNA-seq experiments become available. The expression data in COLOMBOS resulting from RNA-seq data are derived directly from the short read sequences as made available through public repositories, usually in a fastq or similar format. These reads are aligned to the reference genome for the relevant species (see Table 1) using Bowtie (23), and counts for each gene are then summarized using HTSeq-count (see Supplementary Materials for details). We performed two experiments with the exact same RNA samples using both microarrays (Affymetrix *E. coli* Genome 2.0 Array) and next generation sequencing (Illumina Mi-Seq) technology to show the validity of our approach (see Supplementary Materials). The experiments have been deposited in GEO and are available as GSE48776 and GSE48829, respectively.

## FUNCTIONALITY UPDATE

### Web interface redesign

The web interface tools of COLOMBOS are all constructed around the concept of a (gene expression) ‘module’. A module is the result of a query to the database and contains expression data for a set of selected genes and a set of selected condition contrasts. The original COLOMBOS (v1.0) interface had several query options, but these were spread across different pages and required the user to click through multiple screens to select all the options before launching a query. The query interface and functionality have now been completely redesigned to better accommodate the most frequent query type: a prominent ‘Quick search’ option has been introduced where users specify a (set of) gene(s) of a given organism and do not need to provide any further input to create a module. A diverse set of flexible search functionalities is now contained within a single ‘Advanced search’ option, which allows users to explicitly control the selection of the two dimensions that define a module, i.e. genes and conditions, based on their annotation or expression behaviour. The ‘Advanced search’ also features a number of commonly employed complex operations, which were previously only available after creating a module but can now be specified directly before launching a query, such as clustering the module genes in sets of co-expressed genes or finding additional co-expressed genes in the entire compendium.

Once modules have been created they are retained and can be organized in a user workspace. From there, they can be visualized, analysed or edited further (removing or adding genes or contrasts). Visualization of the created modules, which was previously limited to an interactive heatmap, has now also been extended to include fully interactive and configurable network representations that visualize the relational interactions that exist between the module genes and their available annotation, such as transcription factor regulation, pathway information or transcription unit assignments. COLOMBOS also supports a true multi-query approach in its analysis tools, as multiple modules can be operated upon and visualized simultaneously.

### Programmatic access

The COLOMBOS database can now be programmatically accessed and queried through a REST web service, so that external resources can include our expression data in reports that they generate for their users. This REST web service contains an extensive API with a myriad of functions to list and query the database content. The output of these operations is provided in JSON format to allow other web resources to easily integrate the results into their own site. More information on the options and usage of this web service can be found within the help documentation on the COLOMBOS website. As a proof of concept for the feasibility of programmatic access to the data through the REST API, we used it to develop an R package (made available through CRAN: http://cran.r-project.org/web/packages/Rcolombos/). This R package allows users to perform complex queries to the database from within the R statistical environment and take advantage of the huge collection of R packages to perform further statistical analysis and visualizations.

## DISCUSSION AND FUTURE PLANS

COLOMBOS aims to be the prime database for bacterial genome-wide expression data, whether by providing microbiologists a convenient resource to complement their in-house research, or by providing researchers in systems biology with the valuable asset of large-scale expression data. As new experimental data are made available, updated versions of the expression compendia will continue to be released in a yearly fashion. The inclusion of RNA-seq data into our compendia is in this regard a major aspect in our commitment to further develop and expand this database. We additionally aspire to keep an open dialogue with our users and plan to add additional prokaryotic species as interest arises.

One of the main strengths of COLOMBOS remains the uniform, clear and computer accessible condition contrast annotations that have been assigned to all the experiments available in the database. While efforts have been made to improve the MIAME (and now MINSEQE for next-generation sequencing) reporting standards for the description of the tested biological conditions, the consistency of sample annotation in public repositories remains an issue, as was highlighted in the most recent GEO update article (2). The COLOMBOS condition description maintains its consistency by careful manual curation, annotating every imported experiment into a set of formal condition properties. The condition property terms assigned to each condition are hierarchically linked through two separate trees: the lower level being a custom tree describing the type of biological property (e.g. mutation, growth medium additive, etc.). The second, higher level is a ‘condition ontology’, which relies on the same terms as the gene ontology (GO) biological process subtree (24) and maps the condition properties used to annotate the condition contrasts to one or more biological processes or functionalities they most likely affect. The combination of a simple descriptive tree and a more complex but widely used hierarchical structure as GO makes the annotation highly intuitive for any life scientist. COLOMBOS’ annotation system is currently being revisited in an ongoing joint effort with the curators of RegulonDB (17), to create a unified vocabulary between the COLOMBOS ontology and the growth conditions as described in the literature available in RegulonDB. At the time of writing around one-fourth of the COLOMBOS condition annotation terms for *E. coli* have been unified between RegulonDB and COLOMBOS.

The massive expression collection of different bacterial species contained within COLOMBOS has already allowed the cross-species comparison of the expression behaviour of model prokaryotic species (7,25). Such analyses can provide valuable insight into the evolution of transcription and its regulation among prokaryotic organisms. One of our main focuses for the future will be to make these types of cross-species analysis directly available through the COLOMBOS web interface and programmatic access tools.

## SUPPLEMENTARY DATA

Supplementary Data are available at NAR Online, including [26–40].

## FUNDING

Research Foundation – Flanders (FWO-Vlaanderen) [G.0903.13N to P.M. and K.L., G.0413.10, G.0471.12 and G.0428.13N]; the Consejo Nacional de Ciencia y Tecnología (CONACYT) Ph.D. scholarship (to D.L.-T.); the KU Leuven Research Council [PF/10/010 (NATAR)]; Institute for the Promotion of Innovation through Science and Technology in Flanders (IWT) [SBO-NEMOA, and a fellowship to V.L.]; Ghent University [Multidisciplinary Research Partnership ‘N2N’]. Funding for open access charge: CRI Fondazione Edmund Mach, DBC-IG, [ADP P1111030I].

*Conflict of interest statement*. None declared.
